# The effect of levodopa on bilateral coordination and gait asymmetry in Parkinson’s disease using inertial sensor

**DOI:** 10.1038/s41531-021-00186-7

**Published:** 2021-05-14

**Authors:** Minji Son, Seung Hwan Han, Chul Hyoung Lyoo, Joo Ae Lim, Jeanhong Jeon, Kee-Bum Hong, Hoon Park

**Affiliations:** 1 Deepmotion analytics Co., Ltd. Research Institute, Seoul, South Korea; 2 Department of Orthopaedic Surgery, Gangnam Severance Hospital, Yonsei University College of Medicine, Seoul, South Korea; 3 Department of Neurology, Gangnam Severance Hospital, Yonsei University College of Medicine, Seoul, South Korea

**Keywords:** Neurological manifestations, Parkinson's disease

## Abstract

This study aimed to evaluate the effect of levodopa on the phase coordination index (PCI) and gait asymmetry (GA) of patients with Parkinson’s disease (PD) and to investigate correlations between the severity of motor symptoms and gait parameters measured using an inertial sensor. Twenty-six patients with mild-to-moderate-stage PD who were taking levodopa participated in this study. The Unified Parkinson’s Disease Rating Scale part III (UPDRS III) was used to assess the severity of motor impairment. The Postural Instability and Gait Difficulty (PIGD) subscore was calculated from UPDRS III. Patients were assessed while walking a 20-m corridor in both “OFF” and “ON” levodopa medication states, and gait analysis was performed using inertial sensors. We investigated the changes in gait parameters after taking levodopa and the correlations between UPDRS III, PIGD, and gait parameters. There was a significant improvement in PCI after taking levodopa. No significant effect of levodopa on GA was found. In “OFF” state, PCI and GA were not correlated with UPDRS III and PIGD. However, in “ON” state, PCI was the only gait parameter correlating with UPDRS III, and it was also highly correlated with PIGD compared to other gait parameters. Significant improvement in bilateral-phase coordination was identified in patients with PD after taking levodopa, without significant change in gait symmetricity. Considering the high correlation with UDPRS III and PIGD in “ON” states, PCI may be a useful and quantitative parameter to measure the severity of motor symptoms in PD patients who are on medication.

## Introduction

Variable patterns of gait disturbance can be found in patients with PD, including the problems of gait initiation, freezing of gait, reduced balance and postural control, reduced step lengths, increased step times, and slow walking speed^1–3^. Since assessing gait clinically just based on a few items on the Unified Parkinson’s Disease Rating Scale (UPDRS) may not be objective and quantitative^3^, gait analysis could potentially be a promising tool for obtaining objective and quantitative information on the walking behavior of PD patients. Several gait parameters, such as step length, gait speed, and variability, have been used to evaluate the degree of motor impairment and to investigate the effect of levodopa on those parameters in previous studies^1,4–8^.

Gait asymmetry (GA) can be used to assess the contribution of cognitive function to gait symmetricity^9^, and it is a spatiotemporal parameter calculated by step length, swing time, or stance time^10^. The phase coordination index (PCI), which reflects bilateral coordination of gait, is a temporal measure that quantifies the accuracy of antiphase coordination and the consistency of left–right stepping^11^. Both GA and PCI values are higher in PD patients as compared to elderly subjects or healthy adults, since the impaired inputs from basal ganglia to neuronal circuits are manifested in the poor bilateral coordination of gait in patients with PD^9^. In addition, GA and PCI are distinctive from each other. While GA (i.e., left–right swing times) may reflect the degrees of similarity in the motor function, left–right step-phase coordination (PCI) may represent the degrees to which the rhythmic process of stepping with one leg is coordinated with the rhythmic process of stepping with the other one^9^. Those parameters have been used to assess the symmetricity and rhythmicity of gait in PD patients, and seem to be closely related to motor impairments^9^. However, no study has investigated the correlation between the severity of motor impairment and those parameters.

In addition, inertial measurement unit (IMU) systems have been proposed as an alternative tool for gait analysis^12–14^, as they are relatively inexpensive, simple, wearable, and require a relatively smaller amount of laboratory space than more conventional systems, such as optical motion capture systems^15,16^. Several validity studies have compared IMU systems to optical motion capture systems^15,17^, confirming that IMU systems can be easy and reliable means to measure gait events in patients with PD. Curtze et al. investigated the responsiveness to levodopa on variable gait parameters using six inertial sensors in 104 subjects with PD, and found improvements in stride velocity, stride length, and range of motion^18^. However, the effects of levodopa on GA or PCI still remain unclear.

Therefore, the purpose of the present study was to evaluate the effects of levodopa on GA and PCI in PD patients, and to investigate the correlation between the severity of the motor symptoms and those gait variables as measured by IMU systems. We hypothesized that GA and PCI might show higher responsiveness on levodopa than other gait parameters, and could be used as a quantitative indicator to assess the severity of motor symptoms in patients with PD.

## Results

### Change of clinical and gait parameters after the levodopa challenge test

The results of the levodopa challenge test are presented in Table 1. In all patients, the average UPDRS III (*P* < 0.001) and PIGD (*P* = 0.003) significantly improved after taking levodopa medication. The average PCI (*P* = 0.008) significantly decreased after taking medication, and the post hoc power was 0.87 for PCI. There was no significant difference in GA (*P* = 0.123). In the “ON” state, gait speed (*P* = 0.039), step length (*P* = 0.031), and the cadence (*P* = 0.035) were significantly higher than those in the “OFF” state. The SRM analysis revealed that levodopa induced the largest improvement in PCI (SRM = −0.64) among all variables. The UPDRS III (SRM = −0.52) showed moderate response to levodopa. The other gait variables (SRM = 0.03–0.5) showed small responsiveness.

**Table 1:** Comparison of gait characteristics between “Off” and “On” states.

	Off state	On state	*P* value	SRM
UPDRS III score	27.7 ± 12.0	22.1 ± 11.1	<0.001	−0.52
PIGD	5.35 ± 2.19	4.62 ± 1.92	0.003	−0.33
PCI (%)	4.39 ± 1.64	3.33 ± 1.84	0.008	−0.64
φ	179.0 ± 6.6	177.8 ± 2.5	0.409	
φ_CV	2.4 ± 2.8	1.9 ± 0.9	0.380	
φ_ABS	5.4 ± 4.9	3.7 ± 1.6	0.069	
GA	3.21 ± 2.05	2.53 ± 1.49	0.123	−0.33
Numbers of steps	49.6 ± 16.8	42.4 ± 13.3	0.007	−0.43
*Pace*				
Gait speed (m/s)	1.06 ± 0.25	1.12 ± 0.25	0.039	0.31
Step length (m)	0.55 ± 0.12	0.58 ± 0.11	0.031	0.24
Cadence (step/min)	110.6 ± 10.1	115.6 ± 13.4	0.035	0.49
*Rhythm*				
Step time (ms)	515 ± 36	511 ± 34	0.681	−0.11
Step swing time (ms)	409 ± 30	408 ± 35	0.925	−0.03
Step stance time (ms)	620 ± 57	614 ± 42	0.577	−0.12
*Asymmetry*				
Step-time asymmetry (ms)	15.7 ± 12.2	12.7 ± 11.0	0.382	−0.32
*Variability*				
Step-length variability (m)	0.016 ± 0.004	0.015 ± 0.004	0.774	−0.06
*Postural control*				
Step-length asymmetry (m)	0.021 ± 0.030	0.016 ± 0.015	0.509	−0.15

*SRM* standardized response mean, *UPDRS* Unified Parkinson’s Disease Rating Scale, *PIGD* postural instability and gait difficulty, *PCI* phase coordination index, *GA* gait asymmetry.

Values are expressed as mean ± standard deviation.

### Correlation between clinical and gait parameters

The Pearson correlation analysis with Bonferroni correction between UPDRS III, PIGD, and other variables is presented in Table 2. In the “OFF” state, there was no variable correlating with UPDRS III. Interestingly, in the “ON” state, PCI (*R* = 0.641, *P* = 0.001) was the only gait parameter that correlated with UPDRS III. PIGD was negatively correlated with gait speed (*R* = −0.642, *P* < 0.001) and step length (*R* = −0.621, *P* < 0.001) in the “OFF” state. In the “ON” state, PCI (*R* = 0.647, *P* < 0.001) was the only gait parameter that correlated with PIGD.

**Table 2:** Results of correlation analysis between UPDRS III, PIGD, and all parameters.

Variables	UPDRS III				PIGD			
	Off state		On state		Off state		On state	
	*R*	*P* value	*R*	*P* value	*R*	*P* value	*R*	*P* value
Age	−0.140	0.494	0.094	0.649	−0.072	0.727	0.183	0.372
Height	−0.153	0.456	−0.011	0.959	−0.155	0.449	0.034	0.869
BMI	−0.294	0.145	−0.237	0.244	0.057	0.781	−0.001	0.995
H&Y stage	0.399	0.043	0.526	0.010	0.273	0.177	0.441	0.024
Treatment duration	0.029	0.887	0.012	0.955	0.161	0.433	0.114	0.581
LED	0.375	0.059	0.201	0.326	0.419	0.033	0.272	0.179
*Gait parameters*								
PCI	0.454	0.023	0.641	0.001*	0.361	0.076	0.647	<0.001*
GA	0.479	0.016	0.424	0.035	0.439	0.029	0.381	0.060
*Pace*								
Gait speed	−0.500	0.009	−0.375	0.059	−0.642	<0.001*	−0.493	0.011
Step length	−0.492	0.011	−0.356	0.074	−0.621	<0.001*	−0.452	0.020
Cadence	0.030	0.884	0.266	0.189	0.074	0.721	0.086	0.675
*Rhythm*								
Step time	0.118	0.567	0.141	0.491	0.199	0.330	0.268	0.186
Step swing time	0.031	0.881	0.047	0.822	0.246	0.225	0.212	0.298
Step stance time	0.133	0.517	0.189	0.355	0.124	0.547	0.254	0.211
*Asymmetry*								
Step-time asymmetry	−0.135	0.510	−0.097	0.646	−0.290	0.151	0.138	0.511
Variability								
Step-length variability	0.335	0.109	0.162	0.449	−0.012	0.956	0.165	0.440
*Postural control*								
Step-length asymmetry	0.301	0.135	−0.180	0.380	0.238	0.241	−0.194	0.342

*UPDRS* Unified Parkinson’s Disease Rating Scale, *PIGD* postural instability and gait difficulty, *BMI* body mass index, *H & Y* Hoehn and Yahr, *LED* levodopa equivalent dose, *PCI* phase coordination index, *GA* gait asymmetry.

*Significantly correlated after Bonferroni correction at *P* value <0.0029 (0.05/17).

Partial correlation analysis controlling the relevant covariate with Bonferroni correction is presented in Table 3. In the “OFF” state, UPDRS III was found to be significantly correlated with gait speed (*R* = −0.674, *P* = 0.001) and step length (*R* = −0.704, *P* < 0.001). GA and PCI were not related with UPDRS III in the “OFF” state. However, in the “ON” state, PCI (*R* = 0.657, *P* = 0.002) was the only gait parameter that correlated with UPDRS III. PIGD was also significantly correlated with PCI (*R* = 0.653, *P* = 0.002) in the “ON” state. Gait speed (*R* = −0.604, *P* = 0.005) was related with PIGD showing near-marginal significance in the “ON” state.

**Table 3:** Results of partial correlation analysis between UPDRS III, PIGD, and gait parameters.

Gait parameters	UPDRS III				PIGD			
	Off state		On state		Off state		On state	
	*R*	*P* value	*R*	*P* value	*R*	*P* value	*R*	*P* value
PCI	0.398	0.091	0.657	0.002*	0.510	0.026	0.653	0.002*
GA	0.416	0.077	0.439	0.060	0.382	0.106	0.420	0.073
*Pace*								
Gait speed	−0.674	0.001*	−0.506	0.023	−0.697	<0.001*	−0.604	0.005
Step length	−0.704	<0.001*	−0.506	0.023	−0.719	<0.001*	−0.577	0.008
Cadence	0.108	0.650	0.379	0.100	0.137	0.563	0.147	0.536
*Rhythm*								
Step time	0.039	0.869	0.055	0.818	0.308	0.186	0.305	0.191
Step swing time	0.014	0.954	0.036	0.882	0.332	0.153	0.309	0.186
Step stance time	0.042	0.860	0.057	0.810	0.200	0.398	0.219	0.353
*Asymmetry*								
Step-time asymmetry	−0.282	0.229	−0.011	0.964	−0.300	0.199	0.202	0.407
*Variability*								
Step-length variability	0.164	0.516	0.216	0.389	−0.017	0.946	0.292	0.240
*Postural control*								
Step-length asymmetry	0.104	0.661	−0.152	0.523	0.098	0.681	−0.238	0.313

*UPDRS* Unified Parkinson’s Disease Rating Scale, *PIGD* postural instability and gait difficulty, *PCI* phase coordination index, *GA* gait asymmetry.

*Significantly correlated after Bonferroni correction at *P* value <0.0045 (0.05/11).

## Discussion

In this study, we found that PCI markedly improved after levodopa medication and that it was correlated with UPDRS III and PIGD in the “ON” state. Among gait parameters, PCI was the only gait parameter linked to UPDRS III and PIGD in the “ON” state. Previous studies have shown that anti-PD medications can change the gait patterns of patients during walking tasks;^4,5,19^ however, these studies investigated only spatiotemporal parameters, such as gait speed and step length. Bryant et al.^4^ reported that PD patients in the “ON” state had significantly higher gait speeds, stride lengths, and double-support times than those in the “OFF” state. Lubik et al.^19^ reported that gait velocities and step lengths were higher in PD patients treated with levodopa than before treatment. Although the present study identified consistent results for spatiotemporal gait parameters after levodopa treatment, the primary aim of our study involved identifying the changes in PCI and GA after medication. To our best knowledge, this is the first study to investigate the effects of anti-parkinsonian medications on PCI and GA measured by an IMU system.

We selected the shoe-type IMU system for the measurement of PCI and GA, as IMU sensors attached to both lower limbs may increase the detection accuracy of gait sequences, such as the heel-strike and toe-off sequences. In addition, these sensors are relatively inexpensive, simple to use, and are wearable for patients with PD^16^. The shoe-type IMU system used in our study used sensors mounted in the outsoles of shoes beneath the back of each foot, maintaining the stability of sensor positions without hindering movement. In the validity analysis for shoe-type IMU system used in our study and motion capture system, the resultant linear accelerations for 17 patients with PD indicated excellent agreement (ICC: 0.990–1.000) for spatiotemporal parameters, including cadence, step time, and step length^20^.

We found that PD patients showed the largest improvement in PCI after taking levodopa medications. In the “ON” state, PCI showed a high correlation with both UPDRS III and PIGD. Impaired rhythmicity of gait in PD may reflect reduced automaticity and damaged locomotor synergies, and the basal ganglia was thought to play an important role in initiating and regulating motor programs involved in gait^21^. It was found that levodopa therapy could reduce variability in PD, demonstrating the role dopaminergic pathways play in the impaired gait rhythmicity in PD using the dopa challenge test^22,23^. We assumed that levodopa improves PCI by reducing the variability of gait. In addition, although the step length and gait speed had a significant correlation with UPDRS III and PIGD in the “OFF” states, it is unnecessary and dangerous for patients with PD to undergo gait analysis in the “OFF” state in the clinical situation. Our results indicate that PCI can be a more useful indicator of the severity of motor impairments in PD patients during levodopa medications compared to other gait parameters.

There were no significant changes in GA after taking anti-PD medications; furthermore, GA showed no correlation with the UPDRS III and PIGD in both “OFF” and “ON” states. Yogev et al.^9^ reported that no correlations were found between GA and the asymmetry of classic PD motor symptoms. It has been reported that GA reflects the degree of similarity in motor function for leg propulsion on both sides of the body, while PCI represents the degree to which the rhythmic process of stepping with one leg is coordinated with the rhythmic process of stepping with the other leg^24^. Similar to previous studies, our results indicate that GA cannot be used to assess medication response nor the severity of motor symptoms in PD patients.

We observed that step length and gait speed had a moderate correlation with UPDRS III and PIGD in the “OFF” state. However, in the “ON” state, gait speed was associated with only PIGD showing only borderline significance. The UPDRS III is based on the severity of overall dysfunction of motor symptoms in PD patients^25^, and PIGD used in our study was calculated from UPDRS III (arising from a chair, standing posture, gait, and postural stability/pull test)^18^. We assume that PIGD may be more closely correlated with gait parameters than UPDRS III. A further study investigating the relationship between gait parameters and clinical scores closely related to gait is needed.

Gait analysis has received widespread attention as part of an objective and quantitative assessment for examining PD patients. The advances in wearable technology have made it possible for clinics to easily assess gait parameters, such as PCI. Although the foot-positioned IMU systems used in our study are comfortable devices for PD patients, they are still only available in tertiary hospitals equipped with motion analysis equipment. Most devices developed for community-based monitoring are based on the performance of tasks that mimic the neurological examination based on UPDRS^26,27^. In the future, a new generation of mobile devices combined with IMU systems could improve health care by providing gait assessments outside the hospital.

This study had several limitations. First, since our findings were obtained from the acute levodopa challenge test, caution is needed when applying them directly to the clinical situations. But, the levodopa challenge test is known to be an excellent method for patients to obtain the extent to which parkinsonian motor symptoms respond to levodopa by evaluating the UPDRS III and gait^28^. Second, the “ON” and “OFF” medication conditions were not randomized. Although randomizing these conditions would have enabled avoidance of a practice effect, we preferred to ask patients to do the required 12-h pharmacological washout during nighttime hours and perform the evaluation in the morning for logistical and ethical reasons. Third, we only included patients who could independently walk a relatively long distance of 20 m several times. Compared to previous studies, we found lower LED and UPDRS III and relatively higher cadence and gait speed in the included patients. Therefore, our results may only be applied to patients with mild PD. Further research is needed to apply our results in moderate-to-severe PD patients. Fourth, we used the original UPDRS in this study, as many previous studies investigating the gait in PD used the original UPDRS^4,5,7,8,18,29–31^. The MDS-UPDRS is known as the new version of the UPDRS, and is designed to be more comprehensive than the original UPDRS^32^. However, the correlation between MDS-UPDRS and UPDRS was excellent for both total score (*R* = 0.96) and part III subscore (*R* = 0.96) in a previous study^32^.

In conclusion, we identified a significant improvement in bilateral-phase coordination after levodopa administration in patients with PD; however, there were no significant changes in GA. In addition, PCI was a gait parameter highly associated with the severity of motor impairments in the “ON” state, indicating that this index may be a useful and quantitative parameter for measuring the severity of motor symptoms in PD patients who are on medication.

## Methods

### Participant

Patients were considered eligible for inclusion in this acute levodopa challenge study if they were diagnosed with idiopathic PD based on the United Kingdom Brain Bank criteria^25,33^, had mild-to-moderate-stage PD (H&Y stage: 2–3), were currently taking levodopa medications, were able to walk unassisted, and had a Mini-Mental Status Examination (MMSE) of >24/30^34^. Patients were excluded if they presented with any musculoskeletal and neurological disorder, other than idiopathic PD, that could affect gait.

After consideration of the eligibility criteria, 26 patients with PD were enrolled in this study. Baseline demographic characteristics of study participants are presented in Table 4. At baseline, age, sex, height, weight, H&Y stage, MMSE, Montreal Cognitive Assessment (MoCA), UPDRS total score, disease duration, duration of levodopa treatment, and levodopa equivalence dose (LED) were assessed for each eligible patient. Two cognitive screening tests (MMSE and MoCA) were conducted, since the cognitive function can affect gait. MMSE is the primary screening tool and the most commonly used instrument in PD^35,36^. MoCA is considered a better measurement of cognitive status in PD, as it lacks both ceiling and floor effect^37^.

**Table 4:** Demographic data of the included patients.

Variables	Total (26 patients)
Age (years)	71.8 ± 6.7
Height (m)	1.6 ± 0.1
Weight (kg)	64.2 ± 8.5
Body mass index (kg/m²)	24.1 ± 3.1
Sex (M:F)	18:8
H&Y stage	2.2 ± 0.3
MMSE	26.3 ± 3.3
MoCA	23.1 ± 5.0
UPDRS total	35.7 ± 15.2
Disease duration (years)	4.9 ± 2.9
Duration of levodopa treatment (years)	3.7 ± 2.8
Levodopa equivalent dose (mg/day)	450.3 ± 213.7

*H&Y* Hoehn and Yahr, *MMSE* Mini-Mental State Examination, *MoCA* Montreal Cognitive Assessment, *UPDRS* Unified Parkinson’s Disease Rating Scale.

Values are expressed as mean ± standard deviation.

This study was approved by the Institutional Review Board of our institution (IRB No. 3-2018-0095). Each participant provided his or her written informed consent before participating in this study.

### Patient assessment

Gait assessment was first performed in an “OFF” levodopa state and then in an “ON” levodopa state, for each participant on the same day. For “OFF” medication testing, participants were evaluated in the morning after abstaining from levodopa medications for a minimum of 12 h prior to the trial. After “OFF” testing, participants took their usual levodopa medications. The peak of levodopa effect was subjectively determined by patients as the best “ON” state and was thus variable; however, most studies set this assessment period at 1 h^18,29^, knowing that the half-life of levodopa is around 1.5 h^38^. When both the patient and the evaluator agreed with the best “ON” state for gait analysis, the patient then took walking tests for the “ON” medication testing. The mean waiting time after the medication was approximately 1–1.5 h. Each patient underwent two trials of walking tests in each state, and the average values from the two trials in each state were used to increase the reliability of data. After each trial, patients rested on a chair for at least 2 min to avoid the effects of fatigue.

**Fig. 1:**
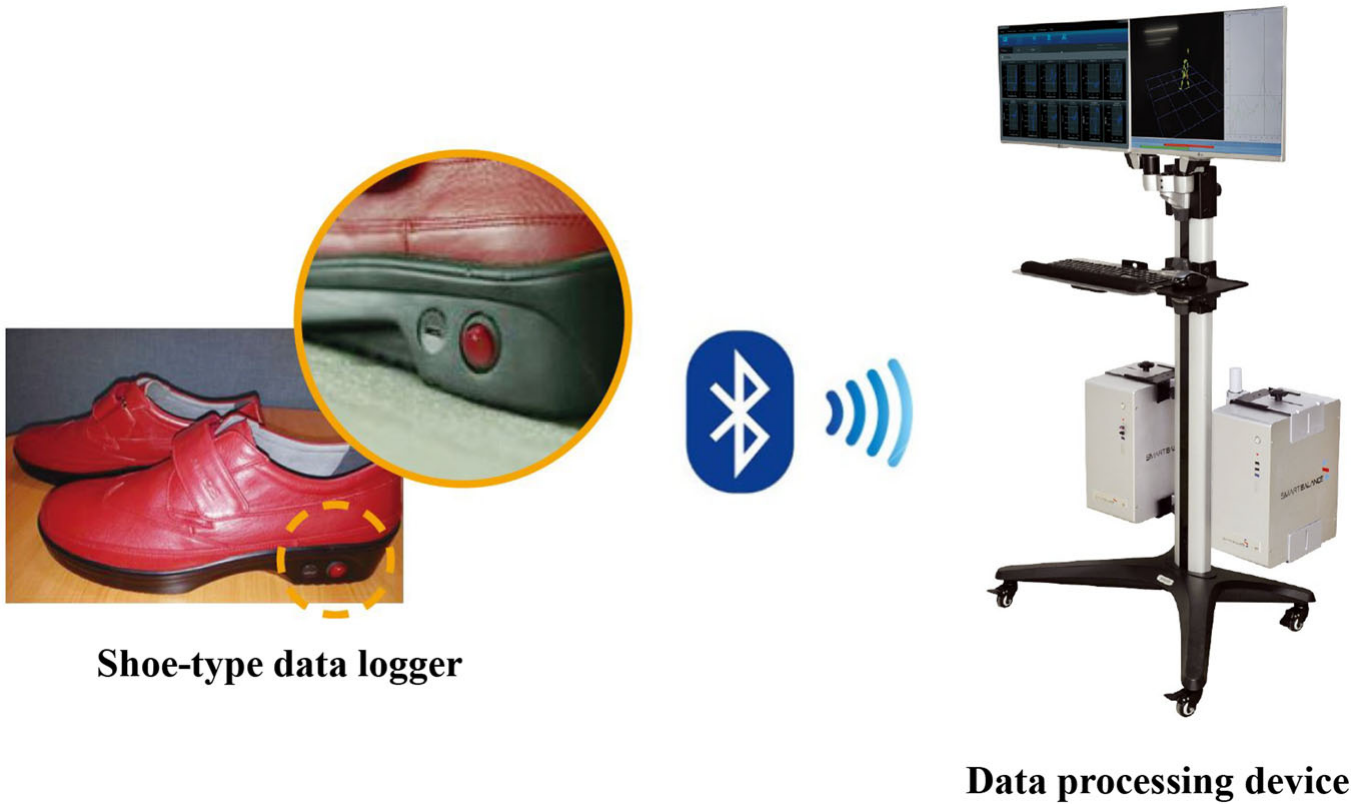
Inertial sensors are mounted on the shoes. Shoe-type data logger can record gait data continuously during walking. The collected gait data is transferred to the data processing device via Bluetooth wirelessly.

For the walking tests, participants were asked to walk forward along a 20-m-long and 2-m-wide corridor at their normal pace. A recent study reported that more than 23 strides were required to obtain a reliable, characteristic PCI value^39^. This means that calculating PCI using gait data obtained by ground walking on a short distance may be unreliable. Therefore, in our study, gait analysis was performed on 20-m-long corridor to collect gait data on more than 23 strides. The gait protocol was performed with an IMU sensor-based gait analysis system (DynaStab™, JEIOS, South Korea) consisting of a shoe-type data logger (Smart Balance1 SB-1, JEIOS, South Korea) and a data acquisition system (DynaStab-Spotfire1, Tibco Spotfire 7.10) (Fig. 1). The shoe-type data logger included an IMU sensor (IMU-3000™, InvenSense, USA) that measured triaxial acceleration (up to ± 6 g) and triaxial angular velocity (up to ± 500° s − 1) along three orthogonal axes^12,16^. The IMU sensors were installed in both shoe outsoles, and the data were transmitted wirelessly to a data acquisition system via Bluetooth®. Shoe sizes were adapted to each participant, with available sizes ranging from 225 mm to 280 mm. The local coordinate system for the IMU sensors included the anteroposterior, mediolateral, and vertical directions.

The UPDRS III was used to assess the degree of motor impairment in all participants. Patients were assessed for their UPDRS III in both “OFF” and “ON” medication states by a neurologist who was blinded to the study hypothesis. The Postural Instability and Gait Difficulty (PIGD) four-item subscore was calculated from the UPDRS III (arising from a chair, standing posture, gait, and postural stability/pull test)^18^.

### Gait analysis

Spatiotemporal parameters were measured using the shoe-type IMU sensor-based gait analysis system. Data on gait speed, cadence, step time, step stance time, step swing time, and step length on each side were collected. The rationale for the selection of gait parameters into the factor analysis was informed by a comprehensive model of gait developed for older adults^40^ and validated in PD^41^. The model comprises five independent domains: pace, variability, rhythm, asymmetry, and postural control^42^.

The PCI quantifies the bilateral coordination of left–right stepping, according to the equations proposed by Plotnik et al.^9^ PCI incorporates assessments of the accuracy and consistency of phase generation, with the step time being used to determine the phase (φ). Normalizing the step time with respect to the stride time defines the phase of the ith stride (φi)^9^. We first calculated the mean values of swing times for both legs. We then used the leg with the higher average swing time as a reference for the gait cycles, and calculated the phase values for the other leg. Therefore, φi was defined as φi = 360° × (tSi − tLi)/(tL(i + 1)−tLi), where tLi and tSi denoted the time of the ith heel strike of the legs with the longer and shorter swing times, respectively^9^. We considered the phase (φ) as the fluctuation about an ideal line of 180° for each step. φ_ABS was the phase-generation accuracy and was calculated as the mean value of a series of absolute differences between the phase at each stride and 180°. Therefore, φ_ABS was defined as φ_ABS = | φi − 180°|^9^. φ_CV was measured as the level of consistency in phase generation across all of a participant’s strides. Finally, PCI was calculated as the sum of the two percentile values: PCI = φ_CV + Pφ_ABS, where Pφ_ABS equaled 100 × (φ_ABS/180)^9^. Lower PCI values indicated a more consistent and accurate phase generation, which is associated with different health conditions, and higher PCI values indicated a more impaired bilateral gait coordination^24^. The range of the mean PCI in healthy young adults known in previous studies was 2.4–3.2^11,24,43,44^.

GA was assessed by comparing the swing times performed by one leg with respect to the swing times performed by the other, according to the following formula: GA = 100 × | ln(SSWT/LSWT) | , where SSWT and LSWT were the mean values of the swing times for the legs with the shortest and longest mean swing times, respectively^9,10^. We calculated the GA using this method, as it has commonly been used in previous studies^45,46^. Using this definition, a value of 0.0 reflected perfect symmetry, and higher values reflected greater degrees of asymmetry^9^.

Step-time asymmetry and step-length asymmetry were calculated as the absolute differences between the mean of the left and the right steps. Step-length variability was calculated using the standard deviation (SD), rather than coefficient of variation (defined as mean/SD × 100), because it provides clarity for interpretation^47^.

### Statistical analysis

At study commencement, we performed an a priori power analysis for the change of UPDRS III, since there was no previous study investigating the change of PCI. A generic inverse-variance method with the fixed-effects model was used to compute pooled estimates of the effect sizes. This analysis showed that a minimum sample size of 26 patients was required to achieve a statistical significance of 0.05 with an 80% power at an overall effect size of 0.58 for the change of UPDRS III, based on five recent studies investigating the change of gait parameters on levodopa challenge test^5,7,18,30,48^. Sample-size calculation was performed using R (version 4.0.3, R Foundation for Statistical Computing, Vienna, Austria). The post hoc power analysis for the change of PCI was performed using a G Power test (Version 3.1.9.2).

All variables were tested for normality using the Shapiro–Wilk test, and none was found to violate the normal distribution assumption. Descriptive statistical analysis using means and standard deviations were used to describe the characteristics of each variable. Paired-sample *t* tests were used to identify significant differences between variables in “OFF” and “ON” states. Responsiveness of the clinical and gait variables to levodopa is expressed as the standardized response mean (SRM). SRM was calculated as the observed mean change divided by the standard deviation of the observed change. SRM is the preferred value to compare paired data measurements at different time points for the same patient. SRM values of 0.8, 0.5, and 0.2 were considered to be large, moderate, and small, respectively^49^.

Pearson correlation analysis was conducted to examine the relationships between UPDRS III, PIGD, and clinical gait parameters. This analysis described the direction and strength of the relationship between two continuous variables in our study, where the direction could be negative or positive. The correlation between UPDRS III, PIGD, and gait parameters was investigated through partial Pearson’s correlations adjusted for age, height, BMI, H&Y stage, duration of levodopa treatment, and LED. Bonferroni correction was applied for multiple comparisons in each analysis. All statistical analyses were performed using SPSS (version 25.0, SPSS. Inc., Chicago, IL). *P* values <0.05 were considered to be statistically significant.

### Reporting summary

Further information on research design is available in the Nature Research Reporting Summary linked to this article.

## Supplementary information

Reporting Summary

## Data Availability

The data that support the findings of this study are available from the corresponding author upon reasonable request.

## Code availability

Sample-size calculation was performed using R (version 4.0.3, R Foundation for Statistical Computing, Vienna, Austria). The post hoc power analysis for the change of PCI was performed using a G Power test (Version 3.1.9.2). Statistical analyses were performed using SPSS (version 25.0, SPSS. Inc., Chicago, IL).
